# Supplementary material for: Are carbon emissions trading and green financial instruments synergistic? -Comprehensive quantitative research based on content analysis
Source: PLoS One. 2024 Mar 7;19(3):e0298601. doi: 10.1371/journal.pone.0298601 (PMC10919601; doi:10.1371/journal.pone.0298601)
Supplement: S1 Data — (ZIP) [file pone.0298601.s001.zip › Data/地级市Z_F工作报告.zip!/░▓╗╒/╕╖╤⌠╩╨/└┤╫╘╣1⁄2╓┌║┼í╛┬φ┐╦╩2╛▌═°í┐_2012.docx]

各位代表：

现在，我代表市人民政府，向大会作政府工作报告，请予审议，并请市政协委员和其他列席人员提出意见。

一、2011年工作回顾

过去的一年，是我市实施“十二五”规划的开局之年。面对复杂多变的宏观经济形势，全市人民在中共阜阳市委的正确领导下，坚持以科学发展观为指导，认真贯彻党的十七届五中、六中全会和省第九次党代会、市第四次党代会精神，以全面转型、加速崛起、富民强市为主线，大力推进经济结构调整和发展方式转变，全面加快工业化、城镇化和农业现代化进程，全市经济社会呈现出速度较快、结构趋优、活力增强、民生改善的态势，较好完成了市四届人大一次会议确定的目标任务，实现了“十二五”良好开局。初步核算，全市生产总值850亿元，比上年增长13%左右；财政收入首次突破百亿元大关、达到115亿元，增长30.5%；固定资产投资400亿元，增长30%；社会消费品零售总额385亿元，增长18%；城镇居民人均可支配收入16500元、农民人均纯收入4950元，均增长18%。

一年来，我们主要做了以下几个方面的工作:

第一，切实把加快发展作为第一要务，全力促进经济快速增长。坚持把做大工业作为经济发展的主攻方向，大力实施工业跨越工程，强化煤电油运保障，先后召开3次工业园区建设与发展现场会，工业经济质量效益同步提高。全年实现规模以上工业增加值290亿元，增长23%，高于全省平均水平2个百分点。工业化率达到35%，提高1.4个百分点。规模以上企业达498户，产值超亿元企业152户，超10亿元企业14户。工业综合效益指数329%，提高30个百分点。加快推进园区建设，全市18个开发园区新增入园企业200多家。阜阳合肥现代产业园区开工建设，首批签约入园企业5家，总投资33亿元。切实加大国有企业改革力度，华源纺织公司改制重组和阜阳轴承公司重组合作工作稳步实施。扎实推进节能减排，除氮氧化物排放量外，其他主要污染物排放和单位生产总值能耗持续下降。深入开展全民创业行动，出台了促进非公有制经济和中小企业跨越式发展的若干意见，全市非公企业和个体工商户分别增加到2万家和13万户。坚持把做强一产作为经济发展的重要基础，扎实推进粮食生产“四大行动”，粮食总产达108亿斤，实现“八连增”。临泉县荣获全国粮食生产先进县称号。大力发展畜牧养殖业，规模养殖比重达到72%，肉蛋奶总量居全省第一。全面启动现代农业示范区建设，省级以上现代农业示范区发展到3个，阜南县黄岗镇入选“全国一村一品示范镇”。市级以上农业产业化龙头企业达304家，年销售收入突破300亿元，增长50%。农民专业合作社发展到1993个，新增621个。坚持把做活三产作为经济发展的重要支撑，大力实施商贸物流提升行动，全市各类专业市场交易额超过600亿元。阜阳临沂商城一期正式开业，香港财富广场主体工程完工，国际汽配城开工建设。大力开展银企对接活动，全年新增贷款98亿元，增长21%。扎实推进金融体制改革，颍东农村商业银行挂牌成立，小额贷款公司发展到22家，融资性担保机构发展到17家。

第二，切实把扩大投资作为有力抓手，强力推进招商引资工作。坚持把项目建设作为扩大投入的重要载体，成立了重大项目谋划编制工作领导小组，分解落实了项目谋划储备任务，全市共编制入库项目419个，总投资2536亿元。坚持市级领导联系重点项目制度，加大项目建设推进力度，全市“6611”工程项目完成投资297.4亿元，占年度计划的115.5%。中航工业开乐万辆罐式专用车、晋煤中能化工合成氨粉煤加压气化技术装置、界首年产万吨铝型材等项目建成投产，昊源集团合成氨原料路线改造、悦康药业头孢类原料药等项目加快推进，淮河流域重点平原洼地治理、沙颍河航道综合整治等项目开工建设。大力构建综合交通体系，阜阳船闸竣工试通航，阜六铁路、漯阜铁路复线电气化改造基本完成。105国道阜阳至太和段建设进展顺利，102省道颍州西湖至临泉段、202省道阜阳至阜南段开工建设。阜阳机场年旅客吞吐量突破20万人次。加大招商引资力度，组织参加了中博会、广交会、徽商大会等重大经贸活动，成功举办了第五届皖西北投资贸易洽谈会、第四届华药会等节会活动。全年引进市外项目1310个，实际到位资金381亿元，增长43%。实际利用外资1.4亿美元，增长29.1%。加大对外贸易力度，进出口总额达6.5亿美元，增长85%。深入开展结对合作，与央企对接合作项目开工14个、完成投资45亿元，中航工业专用车一站式服务平台、开乐罐式专用车三期和国机集团轴承产业园、国家烟草公司阜阳卷烟物流配送中心等重大项目开工建设。民企合作项目开工168个，完成投资215亿元。皖粤经贸合作签约项目66个，总投资580.1亿元。

第三，切实把中心城市作为主要依托，大力加强基础设施建设。坚持把打造阜阳城市经济圈摆上更加突出的位置，启动了新一轮城市总体规划修编工作，编制完成了阜城近期建设规划、土地利用总体规划等专项规划。切实加大阜城建设力度，全年新建改建道路49条、132公里。阜临路、阜王路、人民路改造完成，颍上路泉河大桥、河滨路西段、莲花路东延、南京路南延工程竣工通车，北三环下穿铁路立交工程开工建设，三清路、七渔河路、双清路东段等道路工程加快实施，颍河综合治理等项目前期工作扎实推进。坚持群众利益优先、安置优先，出台了国有土地上房屋和集体土地征收补偿安置办法，开工建设安置小区440万平方米，碧水云天安置小区一期工程交付使用，三角洲东岸和界首路、南京路等安置区加快建设。全年新开工各类保障性住房26100套、基本建成17977套，分别占年度目标任务的116.8%和153.4%。扎实推进城市绿化美化亮化工作，三角洲公园二期、梅园、北京西路绿化和鼓楼广场改造工程顺利完成，西城河景观工程、泉河风景带加快建设。加大城市供水管网改造力度，中水厂项目开工建设，南照淮河取水项目及颍州、泉北污水处理厂前期工作有序推进。全面加强城市管理，积极推行“数字城管”，大力开展阜城交通秩序综合整治，市容市貌得到改善。加大城乡统筹力度，5县市建设步伐加快，扩权强镇试点扎实开展，“百村示范、千村整治”行动计划深入实施，新增省级新农村建设示范村80个。

第四，切实把改善民生作为根本目的，着力发展社会各项事业。坚持把改革成果更多地惠及人民群众，启动实施城乡居民收入倍增计划，财政支出重点向民生倾斜，34项民生工程任务全面完成，累计投入资金55亿元，惠及800多万城乡居民。高度重视就业再就业工作，加强农民工创业园建设，成功举办了第四届返乡农民工招聘洽谈会。城镇新增就业57897人，下岗失业人员再就业16779人，新增农村劳动力转移就业11.4万人，城镇登记失业率控制在4%以内。建立了社会救助和保障标准与物价上涨挂钩联动机制，提高了最低工资及最低生活保障标准。基层医药卫生体制综合改革进一步深化，城乡居民养老保险试点和新型农保试点工作进展顺利，城镇基本养老、基本医疗参保人数分别达到29万人、104万人。深入推进科教兴阜战略，实现高新技术产业产值101.3亿元、增长77%，全年专利授权量达881件，皖北地区首家院士工作站在鑫泉米业公司挂牌成立。积极实施名牌战略，金种子集团“醉三秋及图”荣获中国驰名商标。坚持优先发展教育事业，认真实施阜城中小学布点规划，大力促进义务教育均衡发展，阜阳职教园区、阜阳三中新校区加快建设，阜阳五中顺利组建。深入推进文化体制改革，文艺院团改制和文化市场综合执法队伍组建工作有序进行，建成了103个乡镇综合文化站和800个农家书屋。第三次全国文物普查工作全面完成，第六次全国人口普查工作受到国家表彰。阜南嗨子戏、黄岗柳编和阜阳淮北梆子戏入选第三批国家级非物质文化遗产名录。扎实推进“五城同创”和精神文明创建活动，荣获 “安徽省文明城市”、“安徽省园林城市”和“中国书法城”称号，临泉县、界首市、太和县入选文化部2011-2013年度“中国民间文化艺术之乡”。深入开展“创先争优”活动，刘丽、王现伟荣获第三届全国道德模范称号，任影获得2011年度“三农”人物奉献奖，刘晓琳荣获全国十大健康卫士称号，全市40人入选“中国好人榜”。成功举办了第14届亚洲跳伞锦标赛暨国际跳伞公开赛。持续加强人口和计划生育工作，出生人口性别比偏高的势头得到遏制，低生育水平逐步稳定。老龄和老干部工作进一步加强。妇女、儿童及残疾人权益得到保障。新闻出版、海关、物价、外事、侨务、审计、统计、文物、档案、地方志、地名、扶贫开发等工作也都取得了新成绩。

第五，切实把维护稳定作为重要责任，努力强化社会管理工作。坚持把创新社会管理作为构建和谐阜阳的重要举措，加强和改进新形势下的群众工作。坚持领导干部开门接访、带案下访，一批信访积案得到清理化解。认真开展“大走访”活动，深入进行矛盾纠纷大排查，努力解决群众关注的热点难点问题。进一步加强和改进法律援助工作，完善人民调解、行政调解、司法调解联动的大调解工作体系。强化食品药品、公共卫生、安全生产监管措施，集中开展校车安全排查专项整治行动，安全生产形势总体平稳。加大闲置土地清理力度，制定了阜城土地规划房产历史遗留问题处置意见，努力解决土地征用、房屋拆迁、环境保护等领域突出问题。进一步建立健全应急管理体系，市政府应急指挥中心建成使用，王家坝镇被省政府确定为首批省级应急管理示范点。深入推进社会治安防控体系建设，组织开展“打四黑除四害”专项行动和网上追逃专项督查“清网行动”。大力实施“清剿火患”战役，火灾隐患排查整治深入推进。支持驻阜部队建设，武警、人防、民兵预备役和双拥优抚工作进一步加强。民族、宗教、港澳台、气象、地震、出入境检验检疫等工作也都取得了新进展。

在加快经济社会发展的同时，我们不断加强政府自身建设，修订了《阜阳市人民政府工作规则》，完善了政府法律顾问、重大决策专家咨询等制度。坚持市政府常务会议学法制度，全面启动“六五”普法。深入推进政府信息公开工作，升级改版了市政府网站，开通了网络问政平台和“12345”市长热线电话。扎实开展作风建设年活动，继续进行市直单位百名科长民主考评，认真清理规范性文件和行政审批项目，加强公务用车问题专项治理，强化行政监察和审计监督，政风行风进一步好转。各级政府自觉接受人大法律监督和政协民主监督，认真办理人大代表议案、建议和政协委员提案，广泛听取各民主党派、工商联、无党派人士及各人民团体的建议和意见，鼓励支持人民群众、新闻媒体对政府工作进行监督，政府工作透明度和社会公众参与度进一步提高。

在肯定成绩的同时，我们也清醒地认识到，我市经济社会发展中仍存在一些突出矛盾和问题。一是发展不快、结构不优的问题依然突出，工业化、城镇化水平偏低，与全国、全省平均水平仍有较大差距。二是投资规模依然偏小，招商引资水平不高，新兴产业比重较低，服务业发展相对滞后，非公经济发展不足，部分企业成本上升、经营困难、效益下滑。三是能源、资金、土地、水资源等要素制约日益突出，节能减排和生态环境建设压力越来越大，转变经济发展方式任重道远。四是社会管理和公共服务历史欠账较多，城乡居民收入持续快速增长难度较大，社会热点难点问题和不稳定因素较为突出，安全生产形势仍很严峻。五是发展环境亟待进一步优化，一些部门和工作人员思想解放不够、创新意识不强、工作作风不实、行政效能不高等问题仍较突出，形式主义、官僚主义和奢侈浪费、消极腐败现象还不同程度地存在。对此，我们一定高度重视，采取更加有力的措施，切实加以解决。

各位代表，过去的一年，面对诸多压力和挑战，我们团结协作、奋力拼搏，取得了经济社会发展的新成绩，迈出了全面转型、加速崛起的新步伐。这是省委、省政府和市委坚强领导的结果，是市几套班子大力支持的结果，是全市人民锐意进取、扎实苦干的结果。在此，我代表市人民政府，向辛勤工作在全市各条战线上的广大干部群众，向给予政府工作大力支持与监督的人大代表、政协委员、各民主党派、工商联、无党派人士、各人民团体、新闻媒体和其他各界人士，向关心支持阜阳建设的离退休老干部、老职工，向为阜阳改革发展稳定做出积极贡献的解放军指战员、武警官兵、预备役部队、政法干警，向中央和省驻阜单位及所有关心支持阜阳建设与发展的同志们、朋友们，向在我市创业的投资者、建设者，致以崇高的敬意和衷心的感谢！

二、2012年工作总体要求

今年是实施“十二五”规划承上启下的重要一年，也是推进阜阳跨越转型、加速发展的关键一年。我们既面临着宏观形势偏紧、结构矛盾突出、要素制约明显和区域竞争激烈等困难和挑战，又有着国际国内产业加速转移、中部崛起战略深入实施、中原经济区建设全面启动、皖北振兴力度加大等众多机遇，总体上机遇大于挑战。同时，经过多年积累，我市自主增长能力明显增强，已迈入工业化、城镇化加速推进的新阶段，进入了大有作为的黄金发展期。只要全市上下振奋精神，坚定信心，攻坚克难，开拓奋进，就一定能够夺取经济社会发展的新胜利，当好皖北崛起的排头兵，成为安徽发展的增长极。

今年政府工作的指导思想是：以邓小平理论和“三个代表”重要思想为指导，深入贯彻落实科学发展观，全面贯彻市第四次党代会和市委四届二次全体扩大会议精神，紧紧围绕打造实力阜阳、繁荣阜阳、文化阜阳、和谐阜阳，坚持以全面转型、加速崛起、富民强市为主线，把握“稳中求进、进中求快、快中求好”的工作总基调，着力加快工业化、城镇化和农业现代化进程，强力推进项目建设和招商引资，更加重视扩大内需和发展现代服务业，切实改善民生和促进社会和谐，加快建设有实力、有活力、有竞争力的区域性中心城市。

综合各种因素考虑，今年经济社会发展的主要预期目标是：全市生产总值增长13%左右；财政收入增长18%以上；规模工业增加值增长25%以上；固定资产投资增长30%以上；招商引资增长30%以上；社会消费品零售总额增长17%以上；进出口总额增长30%；城乡居民收入增长15%以上；居民消费价格涨幅控制在4%左右；城镇登记失业率控制在4%以内；人口自然增长率控制在8.5‰以内；完成省下达的节能减排年度任务。

实现上述目标，重点把握好以下五个方面。

（一）坚持科学发展，更加注重总量提升。按照科学发展观的要求，始终把加快发展作为第一要务，坚持讲总量、讲速度、讲人均。围绕做强一产、做大二产、做活三产，坚持工业化、城镇化“双轮驱动”，工业化、城镇化和农业现代化“三化”同步、相互融合，做到以农业现代化为基础、工业化为重点、城镇化为保障，走出一条一、二、三产齐头并进、协调发展的路子。

（二）坚持创新推动，更加注重改革开放。始终把改革创新作为推动发展的强大动力，坚持用改革破解前进中的难题。强力推进国有企业等重点领域改革，加快形成有利于加速崛起的体制机制。大力实施科教兴阜和人才强市战略，更加注重结构优化升级和发展方式转变，坚持改造提升传统产业与培育战略性新兴产业有机融合，加快构建更具竞争力的现代产业体系。进一步扩大对外开放，全面放开规划、建设、投融资等市场领域，以先进的理念、开放的思维、市场的办法，充分利用国内国际两个市场、两种资源，不断拓展区域经济发展空间。

（三）坚持远谋近施，更加注重规划引领。始终把规划作为经济社会发展的龙头和第一道工序，全方位、多层次推进各类规划编制工作，高起点、高标准编制产业规划、基础设施规划和社会事业规划，努力形成完备的规划体系。高度重视规划的科学性、前瞻性和可行性，认真处理好当前与长远、总规与详规的关系，以规划引领项目编制、招商引资和扩大投入。严格执行规划，坚持规划一张图、建设一盘棋，切实维护规划的权威性和严肃性。

（四）坚持项目支撑，更加注重有效投入。始终把上项目、抓投入作为加快发展的主动力，继续做好项目谋划编制工作，善于用政策生项目、用优势引项目、用精力找项目、用实干上项目。坚持大招商、招大商，充分利用各类平台，多渠道开展招商引资。更加注重挑商选资，积极引进符合阜阳比较优势、产业特点的大项目、好项目和种子项目。在充分发挥银行主渠道作用的同时，通过集合票据、上市融资、发行企业债券等方式，扩大直接融资规模。

（五）坚持民生优先，更加注重社会管理。始终把解决民生问题作为经济发展的根本目的，把更多的人力、财力、精力向社会管理和公共服务领域倾斜，全面发展科技、教育、文化等各项社会事业，促进公共服务均等化。进一步加强和创新社会管理，扎实做好新形势下的群众工作，维护社会和谐稳定。

三、强力推进经济社会又好又快发展

围绕实现全年经济社会发展目标，重点抓好八个方面的工作：

（一）坚定不移实施工业突破，着力提升工业化水平。深入实施工业跨越工程，加大投资力度，加强运行调节，促进工业经济持续快速发展。一是做大做强支柱产业。围绕打造千亿元煤电煤化产业，稳步扩大刘庄煤矿、谢桥煤矿产能，促进口孜东煤矿及早建成投产，力争华润电厂二期获得国家核准，扎实做好刘庄煤矿、口孜东煤矿坑口电厂前期工作。支持昊源集团、颍上鑫泰和阜南新天化工公司加快发展，实施晋煤中能集团三期扩建工程，启动阜阳煤基专用化学品基地建设。围绕打造农副产品加工基地，加快颍东食品工业园发展，推进金种子生态产业园建设，支持阜阳卷烟厂调整产品结构，促进福润肉类、文王酒业和沪千、东盾木业等骨干企业做大做强。围绕打造城市矿产产业体系，以界首、太和、颍上等循环经济园为依托，切实加强环境保护，转变发展方式，依靠高新技术改造传统产业，大力发展循环经济。围绕打造装备制造业产业基地，加快开乐罐式专用车三期、国机集团轴承产业园建设，大力发展专用车、农机、通用机械等装备制造业。围绕打造医药产业基地，发展壮大悦康、锦辉、金太阳等医药企业。认真落实促进非公有制经济加快发展的政策措施，鼓励非公有制经济参与国有企业改革，进入市政公用事业、社会事业等领域。实施初创企业、微小企业扶持工程和中小企业成长工程，培育更多充满活力的市场主体。二是大力发展园区经济。组织实施园区建设提升年活动，扩大园区规模，完善基础设施，提高投资强度，提升开发建设档次。全力支持阜阳合肥现代产业园区建设，主动搞好跟踪服务，做好土地征收、基础配套等保障工作，着力打造“三化”协调发展的先导区、城乡统筹发展的现代化新城区。进一步加快阜阳经济技术开发区建设步伐，大力发展优势产业和高新技术产业。推进县域工业园扩区升级，加快乡镇工业集聚区和农民工创业园建设，着力构筑特色经济和产业集群。认真落实扶持农民工返乡创业就业政策措施，加大财税和金融支持力度，放宽市场准入条件，促进农民工投身家乡建设。三是强力推进企业技术创新。进一步加大技术改造力度，重点实施昊源化工航天炉、天能集团年产2400万套极板等51个投资亿元以上的技改项目。大力发展生物制药、新材料、新能源、光伏、电子等高新技术产业。认真落实扶持企业上市政策措施，重点培育贝克药业、即发瑞泰等企业，力争及早上市。加快国有企业改革步伐，推进阜阳轴承、华源纺织等企业改革重组工作。四是扎实做好节能减排。认真按照国家能源消费强度和总量双控制的新要求，执行淘汰和限制类行业企业差别电价政策，强化合同能源管理，坚决淘汰落后产能。严格节能减排目标责任制，加强重点行业和重点领域的节能降耗，突出抓好大气、水体、重金属、农业面源污染防治，推进颍州、泉北污水处理厂及中水厂项目建设，加大城市生活垃圾收集和处理设施建设力度，不断提升污水、垃圾处理运行能力。

（二）持续有力推进城乡建设，着力加速城镇化进程。围绕建设区域性中心城市，推进阜城大建设、大发展，全面加快城乡建设步伐。一是科学规划引领城市发展。坚持以规划为龙头，结合新一轮城市总体规划修编，编制实施城市经济圈发展规划及各类专项规划。高水平规划西湖新区，力争早日开工建设。按照城市发展目标，编制完善县城规划和重点中心镇发展规划，形成中心城市、县城、特色乡镇、新农村四级城乡发展体系。二是扩张规模拓展城市空间。加快阜城三清路网和七渔河路网建设，开工建设八里松路网、职教园区路网，实施东三环路及颍河大桥项目建设，加快北三环下穿铁路立交工程建设步伐，完成阜王路、颍上路等道路延伸工程。高标准推进颍河环境综合治理工程，努力打造阜城商贸金融服务新区。三是加强管理做美市容环境。加快七渔河、一道河、七里长沟和泉河城区段环境综合治理，实施东清河、中清河、西清河治理南延工程，开工建设书法广场、农民工广场、市民广场、刘锜公园、植物园，推进三角洲公园三期、西城河景观带、泉河北岸风景带建设，实施三清路、阜太路和阜临路景观绿化工程。加大小街巷改造力度，切实解决路不平、灯不明、下水道不畅等突出问题。启动南照淮河取水工程，加快城市供水并网进度。加大保障性住房建设和旧城改造力度，建设各类保障性住房19500套，新增发放廉租住房租赁补贴1100户。加快碧水云天项目二期、和谐佳苑、白衣桥、职教园区、双清路、莲花路等安置小区建设。深入开展文明创建活动，积极争创全国创建文明城市工作先进市、国家级园林城市和省级卫生城市、环保模范城区。继续加强交通秩序综合整治，着力改善市容市貌。四是多措并举破解瓶颈制约。依法加强土地房屋征收和补偿安置工作，严格控制和查处违法建设，加快解决土地、规划、房产等历史遗留问题。深入实施土地整理和整村推进，全面清理各类闲置土地，严格规范土地管理，促进集约节约用地。认真落实耕地保护国策，严厉查处违法用地行为。进一步增加商住用地和普通商品房供给，促进房地产业平稳健康发展。五是城乡统筹带动县域经济发展。引导各地发挥比较优势，大力推进工业化、城镇化和农业现代化进程，进一步提升县城综合承载能力，培育壮大一批基础好、潜力大的中心镇，发展特色经济、开放经济和园区经济，做大总量、形成特色，力争实现在全省争先进位的目标。

（三）坚持不懈抓好“三农”工作，着力加快农业现代化步伐。坚持把解决农业、农村、农民问题作为重中之重，大力促进江淮粮仓向江淮厨房转变、农业大市向农业强市跨越。一是增强粮食生产能力。按照“稳定面积、主攻单产、提升品质、增加总产”的思路，继续实施“4412”粮食提升工程，深入推进粮食生产“四大行动”，扎实开展太和、界首小麦千斤县和临泉、颍州玉米千斤县创建活动，努力提高粮食单产和优质化率，力争总产达到110亿斤。二是构建现代农业产业体系。加快农业结构调整，推进土地有序流转和适度规模经营，统筹推进新一轮“菜篮子”工程建设，大力发展特色农业、高效农业，加强阜南杞柳、闻集草莓、界首马铃薯、太和中药材、临泉脱水蔬菜等特色农产品生产基地建设。深入实施农业产业化倍增计划，支持三宝线业、京九丝绸等龙头企业扩大规模、延伸链条、提升档次。加快现代农业示范区建设，重点抓好颍上国家级和临泉、颍泉省级现代农业示范区建设工作，鼓励支持各地争创国家级现代农业示范区。进一步加大农业科技投入，加强基层农业技术推广服务体系建设。实施畜牧业升级计划，突出抓好规模化标准化健康养殖，加快百万吨安全畜产品供给主体区建设，完善动物疫病防控和畜产品质量安全监管体系。深入实施水产跨越工程，推进林业重点生态工程、万里绿色长廊工程建设。三是大力促进农民增收。认真贯彻落实全省农民增收现场会精神，不断拓宽增收渠道，增加农民家庭经营性、工资性、转移性和财产性收入。加快建立新型职业教育培训体系，大力培育新型农民工。推进农民专业合作组织建设，不断提高农业组织化程度。坚持开发式扶贫方针，实施新一轮扶贫开发攻坚。四是加强农业基础设施建设。抢抓国家加快水利改革发展和新一轮治淮工程机遇，深入开展小型农田水利重点县建设，推进平原洼地及中小河流治理，新增、改善除涝面积10万亩。加快实施大中型灌区建设、排灌泵站更新改造和涵闸加固工程，新增、改善有效灌溉面积20万亩。加强农业气象综合监测网建设，提高防灾减灾和信息服务能力。实施农机富民工程，不断提高农业机械化水平。进一步整合涉农资金，加强乡村道路、供排水等基础设施建设，实施农村小康环保行动计划和清洁工程，大力推进新农村建设。

（四）千方百计扩大有效投入，着力增强经济发展后劲。今年初步安排“6611”工程516个，总投资2176.9亿元，年内计划投资340亿元。一是加大项目谋划争取力度。瞄准国家投资导向和产业扶持重点，紧紧围绕“三农”、社会事业、民生工程、战略性新兴产业和重大基础设施建设，进一步调整充实项目库，加强项目谋划争取工作，努力提高项目争取的成功率，力求有更多项目进入国家和省计划盘子。二是加大项目建设力度。坚持市级领导联系重大项目制度，认真落实重大项目部门负责制，加强协调调度，加快重点项目建设步伐。围绕打造综合交通体系，推进漯阜铁路、阜淮三线、阜六铁路、阜新高速、沙颍河航道整治及沿线港口等项目建设，力争商阜杭高铁开工建设。高标准实施市县快速通道建设，及早完成105国道阜阳至太和段工程，加快102省道颍州西湖至临泉段、202省道阜阳至阜南段工程进度，开工建设102省道阜阳至颍上段工程，启动建设308省道太和三角元至界首段工程。实施县乡道路升级改造、农村公路安保等工程，推动公交线路向城区周边乡镇延伸。加快实施阜阳机场通航能力改造提升工程，不断开辟新的航线，力争全年旅客吞吐量突破40万人次。三是加大招商引资力度。组织开展招商攻坚年和会展经济发展年活动，探索改进招商模式，推进领导招商、专业招商、产业招商和以商引商，争取更多的大企业、大集团落户阜阳。积极参加徽商大会等重大招商活动，继续办好皖西北投资贸易洽谈会、华药会和各具特色的节会活动，努力打造品牌，不断提高招商引资实效。四是加大对接合作力度。继续加强与央企、民企和皖粤对接合作，建立健全沟通协调机制，创新服务方式，促进更多的项目落地生根、开花结果。深化与合肥结对合作，力求在园区共建、教育合作、人才培训等方面取得更大的成果。进一步加强与周边地区的联系合作，努力实现优势互补、联动发展。

（五）多措并举打造商贸名城，着力提高辐射带动能力。围绕建设现代商贸物流中心，深入推进商贸物流提升行动，加快发展现代服务业，努力做活商圈、做大市场、做强物流。一是加强商贸市场建设。进一步做好阜阳临沂商城一期活市工作，实施二期工程建设。高标准改造皖西北商贸城，促进香港财富广场早日开业运营，推进颍泉城市商贸服务业发展示范区建设。加快国际汽配城、中航工业专用车一站式服务平台建设步伐，尽快建成阜阳汽贸物流园二期，实施阜阳农产品中心批发市场迁建项目，加快阜阳农机大市场一期招商工作，推进华源医药、烟草、粮食产业等现代物流园建设，规范提升临泉皮革、界首陶庙山羊等生产资料、生活资料市场。二是努力扩大消费需求。继续落实家电下乡等促进消费政策，深入实施 “万村千乡”市场工程和“新网工程”，推进农贸市场改造升级，开展“农超对接”。鼓励支持地方知名品牌产品消费。加强价格监测和市场监管，保持物价总水平基本稳定。三是大力发展现代服务业。认真贯彻全省发展现代服务业大会精神，加快发展现代物流、电子商务、研发设计、信息咨询等生产性服务业，全面提升商贸、餐饮、健身等生活性服务业，积极培育服务外包、动漫、创意、中介服务等新兴服务业。促进旅游业加快发展，支持颍州西湖、颍上八里河、阜阳生态园等旅游风景区完善基础设施，加大宣传推介力度，打响阜阳旅游品牌。鼓励支持发展高档宾馆酒店，提高城市综合接待服务能力。四是努力扩大对外贸易。大力培育外向型企业，壮大人发制品、柳编工艺品和特色食品等出口基地，扩大纺织、服装、特色农产品和调味品等优势产品出口，进一步提升经济外向度。

（六）高度重视财政金融工作，着力发挥经济保障功能。充分发挥财政金融杠杆作用，更好地支持和服务经济社会发展。一是强化财政保障能力。大力培植财源，强化税收征管，确保财政收入较快增长。认真落实积极的财税政策，加强公共财政、国有资本经营、社会保险基金预算体系建设，建立健全政府性债务管理制度。坚持科学理财、勤俭理财，调整优化财政支出结构，严格控制一般性财政支出，发挥财政对转变发展方式、保障和改善民生的支持作用。二是大力提升金融服务水平。进一步加强政银企合作，引导银行机构扩大信贷投放量，力争全年新增贷款130亿元以上。扎实推进金融创新，支持金融机构开发适合中小企业发展特点和个体工商户、农户需求的信贷产品，鼓励县域金融机构将新增存款主要投放在当地，服务实体经济发展。三是加强地方金融体系建设。积极搭建融资平台，支持金融保险机构加强服务网点建设，努力引进股份制商业银行。加快发展村镇银行、金融租赁等地方金融机构，规范发展小额贷款公司、融资性担保机构。深化农村信用社改革，加快推进颍州、颍泉、界首农村商业银行组建工作。加强征信体系建设，营造良好金融生态。

（七）统筹发展社会各项事业，着力构建和谐阜阳。始终坚持以人为本、民生为重，更加注重保障改善民生，全面发展社会各项事业。一是拓展提升民生工程。继续实施34项民生工程，将农村居民最低生活保障补助水平、农村五保户供养补助标准和重度残疾人生活救助标准提高10%，城镇居民医保、新农合财政补助标准由每人每年200元提高到240元。完善民生工程实施和管理机制，加强协调调度，规范资金管理，切实提高工程质量和资金使用效益，不断提升人民群众满意度。二是深入实施科教兴阜战略。大力推进创新体系建设，加大科技投入，设立自主创新和成果转化专项资金，力争全年高新技术产业产值达到140亿元，专利申请量达到2200件。优先发展教育事业，深入实施阜城中小学布点规划，推进义务教育学校标准化建设，大力促进义务教育均衡发展。高度重视学前教育，认真实施学前教育三年行动计划。加快发展普通高中教育，推进阜阳三中、二中新校区建设。大力发展职业教育，实行职业教育市级统筹，加快阜阳职教园区、阜阳技师学院建设步伐，抓好阜阳高等师范专科学校、农业职业技术学院、卫生职业技术学院筹建工作。支持阜阳师范学院创建阜阳大学。完善家庭经济困难学生资助体系，高度重视进城务工人员随迁子女、农村留守儿童、残疾儿童的教育和成长，着力解决学校安全问题。三是大力发展公共卫生事业。深化医药卫生体制改革，巩固和完善新型农村合作医疗制度和基本药物制度，抓好县级公立医院改革试点工作，积极争创“三甲”医院。加快市人民医院和第五、第六人民医院建设步伐，逐步实现基本公共卫生服务均等化。四是加强就业和社会保障工作。加快健全覆盖城乡的公共就业服务体系，多渠道增加就业岗位，力争全年新增就业5.6万人，下岗失业人员再就业1.3万人。加强社会保险扩面征缴，规范养老保险统筹，全面实行医疗、失业保险市级统筹。继续增加企业退休人员基本养老金，完善被征地农民养老保险制度，确保实现城乡居民养老保险制度全覆盖。五是推进文化大发展大繁荣。加强社会主义核心价值体系建设，深入开展社会公德、职业道德、家庭美德、个人品德教育，扎实推进文明城市、文明村镇、文明单位创建工作。加快公共文化场馆建设，规划建设市规划馆、图书馆、博物馆、书法艺术馆和烈士纪念馆，基本实现乡乡有综合文化站、村村有农家书屋的目标。着力发展壮大太和书画、临泉杂技、颍上花鼓灯、界首彩陶、阜阳剪纸等文化产业，努力打造文化知名品牌和文化产业基地。深入推进艺术表演团体和经营性文化事业单位转企改制工作。加强文物和非物质文化遗产保护开发。实施全民健身计划，不断提高竞技水平。尽快建成市体育中心综合馆，努力创建国际航空运动城市。六是加强人口和计划生育工作。认真落实计划生育责任制，突出抓好人口大县、后进乡村和流动人口的计划生育工作。加强利益导向机制建设，综合治理出生人口性别比偏高问题，千方百计提高出生人口政策符合率，稳定低生育水平，确保人口控制目标的实现。

（八）全力维护社会大局稳定，着力加强和创新社会管理。高度重视社会稳定工作，切实提高社会管理水平，不断巩固和谐稳定的良好局面。一是进一步夯实基层基础。推动社会建设和管理重心下移，整合各种资源，不断提高基层社会管理能力。扎实推进社区规范化建设，采取“以奖代补”等方式，加快改善社区办公和服务用房条件。加强农村和谐社区建设，不断扩大农村社区建设覆盖面，持续强化社区自治和服务保障能力。二是加强和改进新形势下群众工作。健全社会稳定风险评估机制，不断完善涉及群众切身利益的重大决策和重大项目社会公示、听证等制度。推进信访工作创新，加大矛盾纠纷排查化解力度。巩固“大走访”活动成果，建立健全联系群众的长效机制。三是加强公共安全体系建设。严格落实安全生产责任制，加大交通运输、建筑施工、非煤矿山、烟花爆竹等重点领域隐患排查整治力度，有效防范和坚决遏制重特大事故的发生。进一步加强食品药品安全工作，规划建设市食品药品检测中心，不断提高食品卫生和药品安全保障水平。深入推进平安阜阳建设，加强社会治安综合治理。强化城乡社区警务、群防群治等基层基础建设，加快完善覆盖城乡的社会治安防控体系。加强流动人口和特殊人群服务管理，综合整治非法集资、黄赌毒等社会问题，严厉打击各类违法犯罪活动，全力维护社会稳定。完善应急管理体制，提高应急处置能力。加强统计基层基础建设，全面推行企业一套表统计制度改革。认真做好新形势下的民族宗教工作，大力发展残疾人、红十字和福利慈善事业，扎实推进养老服务体系建设，切实保障妇女和未成年人合法权益。深入开展双拥和军民共建活动，做好民兵预备役工作，巩固发展军政军民团结。充分发挥老干部在构建和谐社会中的重要作用，支持关工委、老龄委和工会、共青团、妇联工作，切实抓好新闻出版、海关、外事、侨务、港澳台、档案、地名、地方志、地震等各项工作。

四、切实加强政府自身建设

全面完成今年各项目标任务，对政府工作提出了新的更高的要求。各级政府必须切实加强自身建设，着力提升政府工作科学化水平。

第一，进一步强化创新意识，不断提升科学施政能力。各级政府及其工作人员要牢固树立重视学习、坚持学习、终身学习的理念，扎实推进学习型政府建设。认真领会把握中央和省政策精神，主动学习政策、研究政策、用好政策，善于把上级精神、外地经验同本地实际有机结合起来，着力提高研判形势、抢抓机遇和驾驭市场经济的能力。进一步解放思想、更新观念，坚决破除小富即满、小富即安的陈旧观念，以更宽的视野、更新的理念、更高的标准谋划发展、推动工作。大力推进改革创新，学会运用法律和政策手段协调各方利益关系，善于用创新的办法解决发展中的问题。扎实推进事业单位改革，进一步理顺各级政府的事权关系。大力弘扬“王家坝”精神和“农民工”精神，以勇于胜利的坚定信心、攻坚克难的顽强斗志，形成加速崛起的强大合力。

第二，进一步强化法治意识，不断提升依法行政能力。坚持把依法行政作为政府工作的基本准则，严格依照法定权限和程序行使权力、履行职责，管好经济社会事务。切实规范行政执法行为，强化执法监督，推进公正执法、文明执法。严格行政决策程序，完善重大决策事项评估机制，注重决策咨询，凝聚各方智慧，不断提高政府决策科学化、民主化水平。深入推进政务公开，完善政府信息公开工作机制，扎实推进财政预算、公共资源配置等领域的政府信息公开。加大电子政务建设，健全网上办事平台，促进网络问政常态化、制度化，进一步提高政府工作效率和透明度。坚持诚信执政、取信于民，着力打造诚信政府，提高政府的公信力。各级政府要自觉维护党委权威，主动接受人大法律监督和政协民主监督，严格执行人大及其常委会的决议、决定，认真办理人大代表议案、建议和政协委员提案，广泛听取各民主党派、工商联、无党派人士和各人民团体的意见，鼓励支持人民群众、新闻媒体对政府工作进行监督。

第三，进一步强化服务意识，不断提升为民执政能力。坚持把公共服务和社会管理摆在更加重要的位置，真正做到“服务才是管理，为民才能执政”。牢固树立群众观点，始终把群众呼声作为第一信号，群众需要作为第一选择，群众满意作为第一标准，做到思想上尊重群众、感情上贴近群众、工作上依靠群众，与人民群众同呼吸、共命运、心连心。坚持领导干部接访和工作联系点制度，深入开展走访基层群众活动，着力解决群众反映强烈的突出问题，更好地为人民服务、让人民满意。更加关注弱势群体，更加关心群众疾苦，认真解决上学难、看病难、就业难等热点问题，依法保障人民群众权益，让人民群众共享改革发展成果。

第四，进一步强化效能意识，不断提升务实勤政能力。大力开展以“转变作风抓落实、真抓实干促崛起”为主题的落实年活动，坚持办实事、务实效、求实绩，做到真干事、真流汗、真爬坡。进一步转变作风，继续开展政风行风评议和民主考评市直单位百名科长活动。坚持少开会、开短会，少行文、行短文，少评比、评实效。进一步完善目标管理责任考核办法，开展县域经济发展考核评价工作，严格行政问责，切实提升执行落实力。深化行政审批制度改革，进一步清理和减少行政审批事项，规划建设市行政服务中心便民服务大厅，不断改善政务环境。加大经济环境治理力度，大力整治行政不作为、慢作为、乱作为，严厉打击强装强卸、强买强卖、强包工程等扰乱市场秩序的行为，营造公平、高效、有序的社会环境，让投资者放心创业、放手发展。

第五，进一步强化清廉意识，不断提升廉洁从政能力。认真落实党风廉政建设责任制和《廉政准则》，扎实推进惩防体系建设。深入开展反腐倡廉教育，筑牢拒腐防变的思想道德防线。认真落实“一把手四不直管”等制度，加快推进市招投标中心建设，开展廉政风险防控工作，着力从源头上防治腐败。深化预算执行和领导干部经济责任审计工作，加强对重大决策部署落实情况、政府重大投资项目的审计和监督，抓好工程建设等领域突出问题专项治理，坚决纠正损害群众利益的不正之风。认真执行厉行节约规定，严格控制“三公”消费，深入推进节约型机关建设。全体政府工作人员特别是领导干部要始终牢记“两个务必”，严格遵守廉洁自律各项规定，勤勉尽责、秉公用权，当廉洁奉公的表率，做人民满意的公仆。

各位代表，推进阜阳跨越式发展，实现奋力崛起，是时代赋予我们的光荣使命。让我们在中共阜阳市委的坚强领导下，进一步坚定信心，开拓进取，扎实工作，为全面完成今年各项任务而努力奋斗，以优异的成绩迎接党的十八大胜利召开！
